# The Impact of Online Social Networks on Health and Health Systems: A Scoping Review and Case Studies

**DOI:** 10.1002/poi3.97

**Published:** 2015-09-01

**Authors:** Frances Griffiths, Tim Dobermann, Jonathan A. K. Cave, Margaret Thorogood, Samantha Johnson, Kavé Salamatian, Francis X. Gomez Olive, Jane Goudge

**Keywords:** social networks, digital communication, social media, health, health care, pressure group, interest group, self‐help group

## Abstract

Interaction through online social networks potentially results in the contestation of prevailing ideas about health and health care, and to mass protest where health is put at risk or health care provision is wanting. Through a review of the academic literature and case studies of four social networking health sites (PatientsLikeMe, Mumsnet, Treatment Action Campaign, and My Pro Ana), we establish the extent to which this phenomenon is documented, seek evidence of the prevalence and character of health‐related networks, and explore their structure, function, participants, and impact, seeking to understand how they came into being and how they sustain themselves. Results indicate mass protest is not arising from these established health‐related networking platforms. There is evidence of changes in policy following campaigning activity prompted by experiences shared through social networking such as improved National Health Service care for miscarriage (a Mumsnet campaign). Platform owners and managers have considerable power to shape these campaigns. Social networking is also influencing health policy indirectly through increasing awareness and so demand for health care. Transient social networking about health on platforms such as Twitter were not included as case studies but may be where the most radical or destabilizing influence on health care policy might arise.

## Introduction

The ability to access and disseminate information through digital communication networks (e.g., Internet, mobile phones) is changing societal activities, including national politics and election campaigns (Gruzd & Roy, 2014), local politics and activism (Biondo, 2013), and accountability (Sagar, 2013). Within the health domain, there is potential for interaction through digital social networks to enable individuals to interact with others who have similar concerns about health risks or where health care is wanting. This could result in change in health care demand or mass protest (on‐line or off‐line) that impacts on health policy. This article considers whether there are indications that this is happening.

We know that digital communication is changing how people access and receive information about health and health care (Eysenbach, Powell, Englesakis, Rizo, & Stern, 2004), how they share health and health care data including personal experience (Ziebland & Wyke, 2012), and how they collate and interpret these data (Griffiths et al., 2012). There have been examples of social media being used as part of media campaigns for specific treatments to be made available (Pullman, Zarzeczny, & Picard, 2013). People living with chronic illness often become expert at managing their own condition, but not always in the way health professionals expect (Greenhalgh, 2009). Engaging with health‐related digital social networks is one route for gaining this expertise. Although misinformation about health can spread through such networks (Scanfeld, Scanfeld, & Larson, 2010), there is evidence that this is rapidly corrected by participants in the network (Ancker et al., 2009; Armstrong & Powell, 2009; Esquivel, Meric‐Bernstam, & Bernstam, 2006). Interaction through digital social networks can lead to the identification of problems related to health that the professionals have not yet thought about, and to the contestation of prevailing ideas about health and health care. This interaction also has the potential to enable mass protest, where health is put at risk or health care provision is wanting. It is argued that although digitally networked groups, such as mothers of young children and people with rare diseases, are becoming powerful special interest lobby groups, this phenomenon is not replicated across all health issues, population groups, and contexts (Griffiths et al., 2012). However, in the future, the impact of protest about health care provision, engendered through digital social networking, could be greater in countries where accountability of health providers is weak and the health system is inefficient and inadequate. We suggest that digital social networking could provide an innovative approach to enhancing community representation, ownership, and participation in health service policy formulation, as called for by the World Health Organization (WHO): Regional Office for Africa (2012).

In this article, we explore the potential impact of digital social networks comprising people who are not health professionals but who interact about health‐related issues. We are interested in how such networks impact health and health systems and seek to understand how their effect varies in different contexts and why. Our research question is *What is the potential for impact of health‐related, lay‐controlled networked digital communication on health and health systems?*

We are interested in any direct effect on health and health systems at the individual level; for example, where people learn about their health or access health care in a different way as a result of peer interaction on a social network. Policymakers need to take into account such changes in how their populations seek health and health care. However, social networking potentially could have a wider political role. Peer interaction has the potential to lead to political action and influence policy about health and health care. In our case studies we consider the balance between these different activities and their impact on health, health care, and health care policy.

Digital social networking activity may vary in pattern, content, and evolution, depending on the health condition. Similarly, for different health conditions, people engaging with the digital social network may make use of or adapt to the social network in different ways. We focus on health‐related social networks that are initiated and controlled by people who are not part of formal health care systems but who may be interested in health for themselves or on behalf of other people, including society more generally. Network activity can be relatively transient, for example, an exchange on Twitter in response to changes in health care provision (King et al., 2013), or more sustained, such as patients seeking support on how to cope with a specific health problem such as Parkinson's disease (Attard & Coulson, 2012). The networks may be within a wider network context such as Mumsnet (Mumsnet Limited, 2015). Currently, the networks that are most visible are those which have evolved into commercial enterprises, with management teams who may not be health experts but who are experts in a managerial sense.

For this study, we initially include digital social networks where individuals interact with other individuals directly, such as within a discussion forum or on a blog with responses. The interaction may be visible to a limited group of registered users or to the general public. We also include indirect interaction via other individuals (retweets on Twitter are an example), and interaction between an individual and a large group of people (such as occurs on PatientsLikeMe). We identify more sustained and established social networks, given that transient networks that form and disperse quickly are relatively more difficult to capture and study. An example of a transient network would be a thread on Reddit or a Twitter conversation about a health issue. The interaction is transient because of the nature of the platform on which it takes place and the form of the interaction. Most Twitter conversations are between a small set of people but even when large numbers are involved they are still relatively transient. A platform such as Reddit is divided into communities around themes. Examples related to health are fitness and diabetes. Hundreds of new threads can be created daily within each theme. Those that are visible on the front page are those receiving user votes. However, even popular threads do not last more than 1 or 2 days before being buried. With transient social networks it is possible to analyze the overall content using text analysis (e.g., Mishori, Singh, Levy, & Newport, 2014). However, it is difficult to capture sufficient detail of these conversations to understand why they take place, the informational quality, and how and why they may be taken up by other individuals or dropped. Therefore, although we included this type of social network in the initial stages of our scoping study, they were excluded from our case studies.

We explore the impact of sustained health‐related digital social networks as follows. For the scoping review, we establish the extent to which the phenomenon of social networks related to health is documented in the publically available literature, and establish evidence of the prevalence of these networks. In order to select our case studies, we describe the characteristics of documented networks and how they vary. We then select four networks as case studies (Yin, 2009) and explore their structure, function, participants, and impact, seeking to understand how they came into being, how they sustain themselves, and what changed as they matured.

## Methods

Our research uses peer‐reviewed academic literature, other literature including news stories, and examination of social network sites.

### Phase 1: Understanding the Extent to Which Social Networks Related to Health Are Documented and Evidence of the Prevalence of These Networks Related to Health

The following databases were searched: Medline, Web of Science, Embase, and the Applied Social Sciences Index and Abstract (ASSIA) using the keywords: lay, volunteer*, lobby*, pressure group*, interest group*, self help group*, social media, digital media, digital communication, web 2.0, internet, blog*, twitter, facebook, tweet, forum*, crowdsourcing, wiki, email, health, healthcare, medicine, medical. This retrieved 3,154 references after de‐duplication. For this scoping review, we rapidly sorted this literature based on title to exclude irrelevant articles and to exclude, for example, reports of health professionals using social networking as an intervention, or the use of social networking within closed support groups. This initial sift identified 133 potential articles. These were read in full and data extracted on the identity of the studied social network, the research approach used, and a summary of results. News items on social networks were identified using individual newspaper search systems. We excluded non‐English language literature.

### Phase 2: Describing the Characteristics of Documented Networks and How They Vary

From reading the literature and discussion within the research team, we drafted a framework for characterizing the social networks identified in phase 1. We then examined and compared each social network to refine this framework (Table 1). We then re‐examined each social network and summarized its characteristics within this framework (Table 2). Distinct network elements, such as blogs, discussion forums, and multimedia, were easily discernible from the homepage of networks. The dimensions of each network represent the key outcomes from a user's engagement with a particular network. Websites which included considerable informative material (such as explaining more about certain conditions and giving expert advice) permanently embedded within them were classified as active in disseminating information. Where networks had opportunities for person‐to‐person interaction, we considered this as potentially facilitating emotional support and providing guidance. Through our discussions, we decided to distinguish between the spread of established information (text or links to outside sources) versus the collection and collation of information derived from the network itself. We also considered how online network activity between users might translate to wider changes in society and developed dimensions capturing campaigning and fundraising activities. Evidence about network formation came from the published literature or the website itself. The character of each network, such as whether a visible network was present and the degree of anonymity in the network, was deduced by emulating the process of an interested user: accessing certain elements (e.g., a discussion forum), registering a username if required, and exploring the avenues for interaction. Of those we have logged, none had the requirement that users be patients themselves in order to register. In many instances, it is possible to register as a researcher. One anorexia network asked all those registering to either be a current or recovering patient with eating disorders. We, therefore, did not look into it.

**Table 1:** Definitions of the Characteristics of Social Networking Health Information Sites

Components	
Personal profiles	Users have an individual page that can display personal details, interests, friends, photos, likes, etc. This is customizable and the amount of information available to the public is typically user‐defined.
Videos and multimedia	Network has permanently embedded videos or multimedia, which serves to inform or provide emotional support.
Ask an expert	Participants are able to directly contact medical professionals with their health‐related questions through the network website.
Discussion forum	A list of discussion threads, which are user‐generated and in which other users can post replies or comments. Discussion forums are often separated into subgroups or categories (e.g., for specific conditions). In some instances, forums are moderated by professionals.
Blog (expert)	Network hosts articles or blog posts written by medical professionals. This can be to provide either information or advice/tips to users.
Blog/journal (participant)	Users can post their own blog (journal) entries, which are visible to others. Typically, these involve personal reflections, experiences, or advice for others who may read the entries.
Posts/comments	To be distinguished from discussion forums. Posts or statuses are similar in nature to threads but are not structured or categorized by the network owner. They are typically added to a “stream” of other posts made by other users.
Chat/private messaging	Participants within the network have the ability to send private messages (emails), which are only visible to the two interacting parties.
Dimensions	
Dissemination of information	A central aim of the network is the dissemination of established information or advice to users. This may be through permanent text or multimedia, expert contributions through guest articles or blogs, or references to other sources of information.
Collection, collation, and correction of information	To be distinguished from the dissemination of information. This explicitly touches on the emergence (“collection” or “collation”) of information, which is derived from network activity and user contributions within the network.
Emotional support	Classifies networks, which embed elements that support user exchanges of experiences, personal advice, or any other function which serves to promote emotional well‐being.
Campaigning	Through the network, users are active in setting political goals or creating social movements around health issues. Critically, these actions are founded through collective action within the particular network (initiation can be both by owners and users of the network).
Fundraising	The network clearly integrates options for participants to donate or raise money for health‐related causes that are not concerned with the maintenance and operation of the physical network, for example, links or built‐in platforms to donate for charity research.
Network formation	
Medical professional	Network founded by an experienced medical practitioner or “health expert.”
Managerial professional	Network created by an individual with managerial, technical, commercial, or other expertise but which is not associated with expert health knowledge.
Lay	Network formed by individuals who do not possess professional skills that would otherwise be associated with the previous categories. Often these individuals are patients or close to other individuals who have gone through or live with a health condition.
Network character	
Visible network	In networks where users are able to form connections (see below), a visible network means that the social network of each user (for instance the people they follow or friends they have) is visible to other users. Applying this to a macro scale, the list of participants of the network is visible to others.
Subnetwork	This refers to health networks that are embedded within larger, nonhealth‐related social networks, for example, a Facebook group dedicated to raising awareness of cancer.
Formation of connections	Users are able to create “physical” links or ties to other participants within the network. Typically, the formation of a link with another user results in greater sharing of information between the two individuals.
Anonymity	Anonymity captures the extent to which participants can remain anonymous or conceal personal information about themselves. In almost all cases, this is user‐defined: there is an element of choice over how much personal information a user wishes to disclose. Within this characteristic, there are three subclassifications (low, medium, high) which are assigned based on the total amount of information which can potentially be displayed about a user (if they choose to do so).
Accessibility	Accessibility is broken down into the following: (i) the restrictions in place which prevent individuals viewing content on the network, and (ii) restrictions on whether an individual can participate within the network.
Memory	The memory of a network refers to the length of time content is visible in the network. Transient networks (those with very short memories) rapidly update content, with older content pushed down. Within this characterization, permanent memory refers to information or content that is controlled by the owner of the network. In various settings, the memory of a particular piece of content can be influence by user activity (more posts on a discussion thread make it more visible and last longer).
Moderation	Moderation refers to the filtering of user‐created content in the network. This is often done by network owners or experienced users to ensure behavioral guidelines and etiquette are upheld and to prevent the spread of misinformation.
Expert research	This characteristic refers to the use of information derived from activity within the particular network by professionals for research purposes, with the intention of using this information to enhance the experience of users.

**Table 2:**
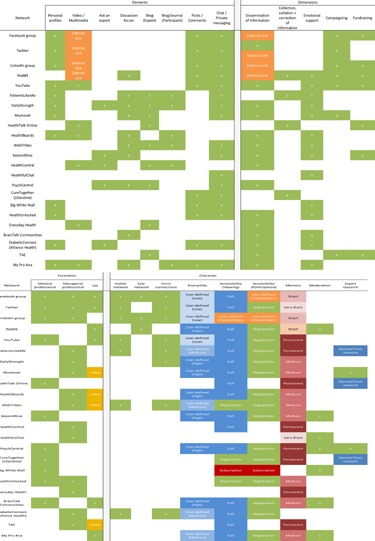
Characteristics of Health‐Related Social Networks

### Phase 3: Choosing and Undertaking Our Case Studies

**Figure 1:**
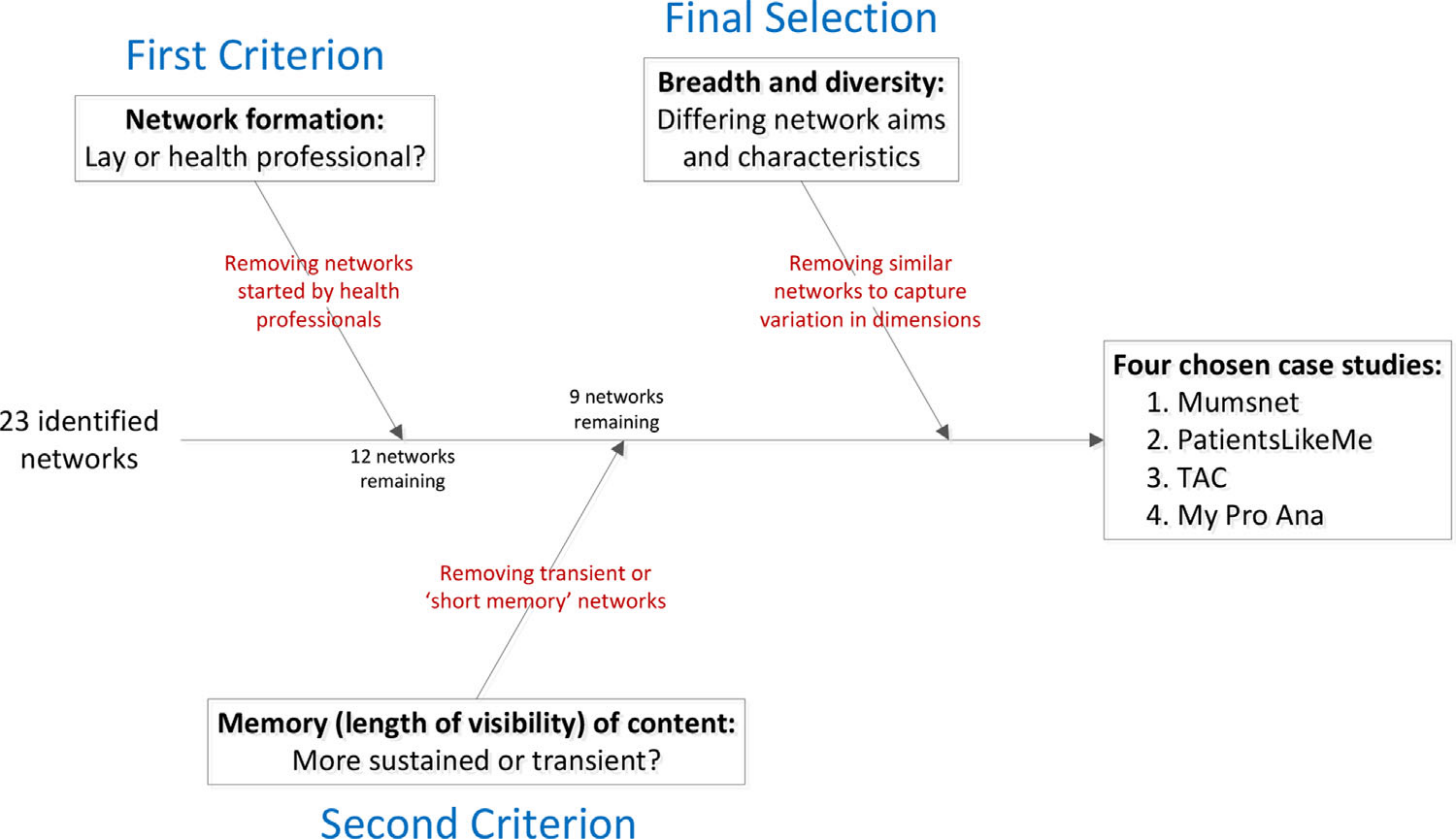
The Case Study Selection Process.

We reviewed the results of phase 2 to identify four diverse case studies. We excluded network sites run by medical professionals. We did not exclude those run by professional managers as this would have excluded the larger more established sites. We then selected from those remaining, four networking sites with different purposes and origins: Mumsnet, PatientsLikeMe, Treatment Action Campaign (TAC), and My Pro Ana (see Figure 1). For each case study, we then searched for relevant literature using ABI Inform and Business Source Premier searching using the four case study site names.

Ninety‐one potential articles were identified. A review of the abstract and full text identified over 30 articles which discussed the history and development of the case studies, examples of their influence in health‐related issues, and articles reporting interviews with key individuals. We also undertook further investigation of the social networks themselves. This included examining site structure, site function (purpose; activity volume; content), participants (local/global; condition specific or not; numbers of new and existing members), impact (evidence of impact on health of participants, on health care services, health care policy, wider issues), how the network came into being, how it sustained itself, and what changed as it matured. Following Yin (2009), in order to provide a structure for our data extraction from the social network sites, we developed propositions about social networks through team discussions. To develop these propositions, we drew on our literature review and the disciplinary knowledge of the research team (Internet science, social science, behavioral economics, epidemiology, clinical/health science, health policy). We developed the propositions to focus our data extraction on our interest in the balance promoted by social network sites of individual level activity related to health/health care versus the formation of politically active groups, including mass protest.

We used these propositions to guide the data extraction and analysis for the four case studies. The propositions were as follows:

1. The structure and function of the social network site impacts on usage and ultimately on sustainability, through the following: (i) quality of user interface; (ii) responsiveness (feedback taken into account); (iii) needs fulfillment (extent to which user preferences can be met); and (iv) security (Harrison, Barlow, & Williams, 2007).
2. The explicit purpose of the site influences the content of social network activity but does not completely limit it (e.g., side conversations can erupt).
3. Volume of traffic (in general or on a specific health issue) of social network sites will determine its impact on health/health care.
4. The nature of the health condition discussed through the social network influences the nature of the social network activity (e.g., sustained use by a stable community of members, people coming and going rapidly).
5. The presence of moderators or established active/expert/respected users influences the impact of social network on individual health but can limit its potential for challenging prevailing norms and knowledge.
6. Social networks influence service provision and health care policy.
7. Condition‐specific content maintains a focus on individual gains from social network and limits the likelihood of the social network influencing community issues such as service provision.
8. Geographically, local networks are more likely to develop campaigns in relation to community issues such as service provision.
9. Lay‐controlled networks that lack professional managerial expertise are not sustained.
10. As social networks mature, they become integrated into the real world network of established social structures (industry, health providers, governments, community, advocacy groups, etc.) and take on attributes and activities of those social structures that have a similar purpose.

## Results

### Phase 1: Understanding the Extent to Which a Social Network Related to Health Is Documented, and Evidence of the Prevalence of These Networks Related to Health

Most of the 133 articles we identified in our literature search reported analysis of content posted on social health networks or reported researcher‐created surveys. Studies included exploring user motivations for participating in such networks, the role of networks in empowering patients, and the influence on the patient–doctor relationship from the perspective of the user. How the social networks were created and maintained was rarely studied, and there was little distinction in the literature between lay‐initiated (or controlled) and professionally managed networks.

Studies commonly focused on a specific network and/or specific health condition. There are analyses of discussion forums dedicated to various conditions including miscarriage (Betts, Dahlen, & Smith, 2014), cancer (Barker & Galardi, 2011; Bender, Jimenez‐Marroquin, & Jadad, 2011; Broom, 2005; Chen, 2012; Huber et al., 2011; van Uden‐Kraan, Drossaert, Taal, Seydel, & van de Laar, 2009), Parkinson's disease (Attard & Coulson, 2012), and eating disorders (Flynn & Stana, 2012; Haas, Irr, Jennings, & Wagner, 2011). These studies monitor network activity over a set period of time, compile scenarios of user interaction, and analyze content for trends. Results indicate that participants tend to seek out networks for emotional support and to find solace from others experiencing similar health problems. For example, a study investigating a miscarriage forum found that users accessed the network to find a “reason for hope,” sharing stories and real life experiences with others to connect for empathic support (Betts et al., 2014). Having experience in common with others in the social network can result in interactions that are less judgmental than in other social arenas. Individuals are willing to openly discuss conditions that are socially very sensitive or embarrassing, for example, on an online men's eating disorder forum (Flynn & Stana, 2012). Where a health issue is very personal in nature, a study of the social networking site EverydayHealth suggests that interaction with lay‐people or other patients may be more influential in inspiring healthy behavior than discussion with medical professionals (Abrahamson & Rubin, 2012).

The quality of information circulating within these networks was studied. We report on studies that focus on the participants' perception of informational quality and exclude studies where authors formally analyzed the quality of information shared. Articles detailing how users perceive quality (Ancker et al., 2009; Armstrong & Powell, 2009; Slaughter, Keselman, Kushniruk, & Patel, 2005; Vennik, Adams, Faber, & Putters, 2014; Williams, Huntington, & Nicholas, 2003) show that many individuals acknowledge that posted information may be from nonexpert sources. Individuals enter such networks to establish a broader understanding of a condition, what it is like living with it, and to seek further details to satisfy their own needs, while having reservations about the source of the information. When users look at network credibility (whether they can trust the information they read), they do so—imperfectly—in various ways, including looking at content comprehensiveness, website complexity, and personal knowledge of the source (Kravitz & Bell, 2013). An analysis of a breast cancer discussion list found that it was considered in the community interest to correct misinformation, with false claims often being corrected quickly (Esquivel et al., 2006). While most studies focused on the perspective of the network user, several also detailed the motivations behind those who created or actively moderate networks. A survey across patient moderators in various online support groups revealed that creators felt that no existing provision accommodated people with the particular health condition, that they wanted to help educate those living with difficult diseases or conditions, and ultimately that they wished to ensure patients did not feel isolated (Coulson & Shaw, 2013).

Nonpeer‐reviewed literature provided stories behind a user's experience within a network and their motivations for participation. It also reported the circumstances that prompted founders to establish these networks. An article linked the founding of the health community PatientsLikeMe by brothers Ben and Jamie Heywood to the diagnosis of their brother with ALS (amyotrophic lateral sclerosis, also known as Motor Neurone Disease) (Independent, 2011), and another described how people living with chronic diseases seek online communities to connect with others and relieve the day‐to‐day stress of their condition (Miller, 2010).

Table 2 lists the social networks identified through this literature review and other well‐known sites identified by the research team.

### Phase 2: The Characteristics of the Documented Networks and How They Vary

The dimensions of the networks identified are summarized in Table 2. It was straightforward to identify most characteristics. When considering formation of connections, we were not concerned with type of element used. For example, the connection could be made through replying to a post on a discussion thread or “commenting” on a Facebook status. When categorizing the levels of “memory” in a network, we viewed the network as a whole rather than considering individual posts or components, although memory (the visibility of information or content) is longer in popular or highly active content.

### Phase 3: The Case Studies

Of the 23 networks identified, only eight did not have formal medical professional input. From these, four case studies were selected. One network, PatientsLikeMe, focused on the collection, collation, and correction of information derived from the network itself. This is an important distinction because almost all other identified networks emphasized the dissemination of existing information. Two had clear campaigning elements attached to them: Mumsnet and TAC in South Africa. TAC was identified from local knowledge, as it represents a network that started as a face‐to‐face network and that later developed a Web presence. The fourth case study, My Pro Ana, claims not to be pro‐anorexia but its content includes participants encouraging others to skip meals and fast. We selected this network as its content is not in line with normal health care advice.

Below, we describe each case study in terms of its structure, function, participants and impact, how it came into being, how it is sustained, and what has changed as it matured. In Table 3, we summarize the evidence to support or not support each proposition. Our description of each case study is based on our examination of the case study network sites, supported by reference to relevant literature.

**Table 3:** Summary of Case Study Findings for Each Proposition

Proposition	PatientsLikeMe	Mumsnet	TAC	My Pro Ana
The structure and function of the social network site impacts on usage and ultimately on sustainability.	Yes. “Virtuous cycle”: high quality user experience and relevant personalized feedback—high volume users providing data—commercially viable	Yes. High quality user experience with relevant information, high user numbers, sustainable as commercial venture.	No. Structure of the website is largely static. The social network is largely sustained by interaction beyond the website.	Yes. Anonymity and ease of interaction (discussion threads) promote activity (Gavin, Rodham, & Poyer, 2008; Rouleau & von Ranson, 2011).
The explicit purpose of the site influences the content of social network activity but does not completely limit it (i.e., side conversations can erupt.)	No. Purpose directs and completely constrains activity. No facility for side conversations.	Yes. There is no evidence that conversations not relevant to Mumsnet's aims are removed.	N/A (no direct social network activity)	Yes. The stated purpose (supporting for those experiencing eating disorders) frames the discussion, but a central focus (proliferation of eating disorders as implied by the name Pro Ana) is not limited by site's explicit purpose.
Volume of traffic (in general on a specific health issue) of social network sites will determine its impact on health/health care.	Potentially yes. Claim that the high volume data will enable medical innovation to improve health (Frost et al., 2011). No examples of success available except one where an intervention was shown not to work as claimed. Unclear if this proposition could be proven even in the future unless commercial companies buying the data released the evidence.	Yes. Evidence that Mumsnet monitors both volume and content of posts to decide on political campaigns, future content and advertising.	Yes, albeit volume refers to on‐the‐ground activity. This is because TAC is largely a political / campaigning oriented network which requires high involvement and awareness to be successful.	Yes but only via individual behaviors. The activity on the site seems to encourage individuals in behaviors that arguably go against mainstream health care (Boero & Pascoe, 2012). There is no evidence of impact beyond individual behaviors.
The nature of the health condition discussed through social network influences the nature of the social network activity (sustained use by stable community of members, people coming and going rapidly.)	Social network activity is constrained by the design of the site—peer‐to‐peer sharing is indirect.	Yes. The social network activity on the site and its topic (being a parent) both touch on all aspects of life (Pedersen & Smithson, 2013).	TAC initially formed through networked groups of HIV/AIDS activists. Large issues—social stigmatization and medicine costs—attributed with HIV/AIDS influenced network campaigning activity (Grebe, 2011).	Yes. The topic (eating disorders, with specific emphasis on supporting pro‐anorexia) attracts a specific type of user. This type of user is more likely to represent an unorthodox view over the health issue, but one which forms consensus within the network itself (Boero & Pascoe, 2012). It is not clear how this impacts on people leaving or joining the network.
The presence of moderators or established active/expert/respected users influences the impact of social network on individual health but can limit its potential for challenging prevailing norms and knowledge.	No moderators or established experts except via ‘Crisis’ section which provides hotline for users.	Yes: several channels for “experts” to influence individual health/well‐being. There are challenges to prevailing norms through the Mumsnet campaigns.	N/A	Unlikely. There is network moderation of discussion threads, but most conversations seem to flow freely. The website claims only to filter out spam or unrelated content. It makes no claim on the role moderation plays in ensuring the medical accuracy or legitimacy of statements made within the network.
Social networks do influence service provision and health care policy	No evidence of any direct influence on service provision and health care policy	Some evidence of successful campaigns related to maternal and child well‐being	Campaigning influences which services should be prioritized or improved.	The controversial nature of the content on such websites has prompted movements to establish policies aimed at banning such networks or limiting their online presence (Chesley, Alberts, Klein, & Kreipe, 2003).
Condition‐specific content maintains a focus on individual gains from social network and limits the likelihood of the social network influencing community issues such as service provision.	For users, the content and structure of site limits gains to individual gains. Influence on service provision is potentially possible but only indirectly via commercial companies buying the data and using it for innovation in health care.	The main focus is on individual gains from the social network. However, the campaigns that are taken up by Mumsnet do seem to be on issues identified in posts to the site but with active monitoring and some intervention from site owners. Mumsnet members also take on campaigns and report on them through Mumsnet.	No. Purpose is improving community provision of treatments.	Yes. Focus is on an individual experience or issue, with the end or outcome being personal emotional relief.
Geographically, local networks are more likely to develop campaigns in relation to community issues such as service provision.	No facility on site that would enable development of campaigns.	This is a U.K. network. Mumsnet campaigns are U.K. centered (although their “guest campaigns” may be international—text and links provided for users).	Yes. Targets local issues by bringing together users within a specific locality (sub‐communities in South Africa)	No. The network is not local and appears to attract users from a variety of different countries (Alexa Internet, 2014).
Lay controlled networks that lack professional managerial expertise are not sustained.	Yes. This site has professional managerial expertise and is sustained.	Yes. This site has professional managerial expertise and is sustained	Yes. TAC has functioned as a large activist organization, which has enabled it to have a much wider impact and sustain itself as a leading campaigning body in South Africa.	Conflicting evidence exists. Niche conditions lead to such networks forming tight lay communities with network‐specific traits which are sustained (Adler & Adler, 2008). However, the frequent disappearance or dormancy of such niche (negative) health networks—such as several case study networks in Haas et al. (2011)—showcases that it becomes difficult to sustain overtime.
As social networks mature they become integrated into the real world network of established social structures (industry/health providers/governments/community and advocacy groups, etc.) and take on attributes and activities of those social structures which have similar purpose.	Little integration with established social structures for provision of understanding of the experience of illness and treatment except the provision of a crisis hot line.	Yes. Campaigns, social networking, and commercial aspects are all integrated with real world network of established social structures.	To be seen. Almost the reverse direction: TAC has emerged out of established social structures into a social network.	No. Socially sensitive and stigmatized conditions such as pro‐anorexia are unlikely to influence or mature to affect real world networks as participation and involvement is held quite secretive (Gavin et al., 2008).
	Yes. This site has become integrated with health‐related industry for the production of innovation in health care. Although providing a novel data collection conduit, the activity of collecting data about what happens as disease progresses and treatments tried is conceptually similar to medical research activities.			

### Case Study 1: PatientsLikeMe

Two brothers founded PatientsLikeMe in 2004 as a result of their experience supporting a close family member suffering from ALS. Their belief was that by creating a network or platform for individuals to share their experiences, patients would gain support and researchers could use contributed data to accelerate the development of treatments. PatientsLikeMe was restricted to only those with ALS until it expanded in 2011 to individuals experiencing any condition. Currently, it claims over 250,000 unique users covering over 2,000 different conditions. The user interface is of high quality. To participate within the network, users create personal profiles highlighting their health conditions and any symptoms they have been feeling. Once a profile has been created, the network automatically links users (via a chart that aggregates data) to others who are experiencing similar problems. Site members can observe how similar or different their experience is from others with a similar health condition.^1^ The aggregate data are continuously updated based on symptoms reported by users each day. PatientsLikeMe advocates for open sharing of health data for speeding up the development of treatment development. It suggests that it can play a role in emerging “patient experiments” where patients initiate studies, monitor their disease‐related symptoms, and pool their data (Frost, Okun, Vaughan, Heywood, & Wicks, 2011; Wicks, Vaughan, & Heywood, 2014). It claims that over 50 published research studies have used information generated through the network. PatientsLikeMe finances its operational costs through the selling of data to its partners, which include pharmaceutical companies and medical device makers. The site provides a Crisis section including a hotline for users and advice about contacting their usual doctor. It does not allow advertising.

### Case Study 2: Mumsnet

Mumsnet was launched in 2000 by a mother who aimed to create an advice‐based website for parents. Although it covers all aspects of parenting, it hosts many health‐related discussions. Overall, it has a following of over 4.8 million unique monthly visitors to its website. While predominantly U.K.‐centric, in recent years, Mumsnet has begun to attract a wider international audience. The components of the network are themselves quite simple: static information and references to material beyond the site, an active discussion forum, blog, and user reviews. Users interact within thematic discussion forums. They can search for retail products relating to parenting in order to view reviews generated by other network users. Mumsnet also hosts an active bloggers' network which links the blog posts of over 5,000 Mumsnet users to one common source. Mumsnet has developed a political campaigning role. Posts to Mumsnet forums and blogs demonstrated discontent with miscarriage care and breast feeding facilities. Groups of individuals formed around these issues and became campaign groups which achieved impact on health care policy in the United Kingdom. Examples of campaign activities on miscarriage care include working with National Health Service (NHS) hospitals to improve local care, contributing to clinical guidelines, and lobbying Members of Parliament. These activities have led to a change in care provision in some NHS hospitals and a number of political parties included miscarriage care improvement in election manifestos. Mumsnet also provides links to external national and local campaigning efforts.

### Case Study 3: Treatment Action Campaign

TAC was first launched in late 1998 as an on‐the‐ground campaign group for improved treatment access for HIV/AIDS in South Africa. This has been a very successful campaign but the majority of network activity occurs offline. Currently, the TAC website is static, providing information and displaying links and contact details to further become involved within the network. There are no networking facilities. This may reflect the poor penetration of Internet access until recently in South Africa. Mobile phone usage is, however, common in South Africa, but there is no evidence from the website of the role of mobile‐based forms of communication such as text messaging in the Campaign.

### Case Study 4: My Pro Ana

My Pro Ana lists itself as an online forum and community to support users afflicted with eating disorders. Formally, the community denies any role in fostering or encouraging those with eating disorders. Little information about the history of My Pro Ana, or indeed networks of a similar nature content‐wise, is available on the website itself. The earliest traceable date for this site is 2013. This might reflect the short lifespan of relative niche (and often negative) health communities, either due to dormancy or forced closure, though more formal investigations are still needed in this regard. The site has a standard set of components that encourage users to share information or discuss their experiences. This includes various thematic‐grouped discussion forums, a live chat log, and gallery for images of inspiration (or “thinspiration” as dubbed by users within the anorexia community). In total, My Pro Ana hosts approximately 115,000 members. Although a majority are from the United States, participants are drawn from across the world (Alexa Internet, 2014). At any given moment, the community is quite active, having at most times 1,000 users online. In terms of topics, the community is split into a variety of sections covering emotional support, physical exercise, diets, and pro‐anorexia behavior. It also has a Twitter presence with almost 2,000 followers. Our examination of posts on My Pro Ana suggests that considerable activity relates to the sustaining of eating disorders. We traced an online petition asking for My Pro Ana to be shut down (change.org, 2014).

## Discussion

This scoping review and the related case studies has aimed to understand the potential for health‐related, lay‐controlled networked digital communication to have an impact on health and health systems, and so act as a driver for policy. In particular, we were interested in whether digital social networking had its impact through influencing individuals' health and health care seeking, with policy‐makers needing to pay attention to this effect. Or, whether social networking generated more general political activity, including mass protest, that has resulted in influence on policy.

Our case studies suggest interaction through social networking sites related to health has the potential to link people who have a health experience in common and who would otherwise not interact because they are geographically isolated from each other (e.g., uncommon conditions), because they are limited in their ability to interact socially (e.g., parents of small children and people with disabling conditions), or because interaction about their health condition is stigmatized (e.g., anorexia nervosa) (Rouleau & von Ranson, 2011). Most interaction on the social networking sites involves individuals seeking peer support as they struggle with their health condition or with managing their parenting role (Plantin & Daneback, 2009). There is evidence from previous research that individuals may gain in terms of emotional support, and learning how to live with their condition. This includes how to access specific treatments. A change in what people expect from health care services and their confidence in demanding treatments and other services is likely to impact on health care policy and provision. Two case study sites (PatientsLikeMe and Mumsnet) claimed to have been established as a response to the difficult experiences of the founders, and are based on a desire for support in their situation. These have become established sites. My Pro Ana claims to support those with anorexia, although its content (similar across other pro‐eating disorder social networks) seems to promote the condition (Haas et al., 2011; Rouleau & von Ranson, 2011). It is relatively new and is a small, condition‐specific site.

On three case study sites, there is evidence of more general activity that aims to change health systems (TAC, Mumsnet, and PatientsLikeMe). The level of control by the digital platform owners over what issues are identified for campaigns and how campaigns are supported varies. PatientsLikeMe keeps complete control as they aim to change health care through selling data for research. There is evidence on Mumsnet of its members taking forward campaigns as individuals or groups and reporting back through Mumsnet, and some of the campaigns appear to be based on the concerns expressed in Mumsnet posts (Pedersen & Smithson, 2013). TAC started as an offline social network and has not yet established active online social networking. Research on TAC campaigning suggests that in addition to the direct effect of the campaign, individual behaviors in relation to HIV in the context of such active political campaigns can contribute to change in social attitudes (Levy & Storeng, 2007). This suggests a further potential indirect route of influence for social networks on policy; that is, individuals aware of the campaign gain confidence in demanding health care. All activities of the social networks directly aiming to change health systems have become integrated with established social structures and social systems rather than giving rise to a new form of mass protest. The content of My Pro Ana is arguably anti‐established health care, but actions taken are by individuals in relation to their own health and not as a social grouping.

### Limitations of the Study

As passive observers of the case study sites, the only evidence available to us on the level of moderation of posts on the site was the published site policy. Our study is limited to English language sites and related literature. However, many non‐English speaking areas of the world are at least as engaged in social networking in relation to health. For example, several Vietnamese online newspapers include a health section with forum for posting or blogs with comments posted.^2^

Using our study approach, we were unable to study transient network interactions on health issues. Understanding how transient interactions, such as on Twitter or Reddit, influence health or health systems is likely to require both online data collection and offline methods, for example, the ethnographic approach used to study parents of children with genetic conditions (Schaffer, Kuczynski, & Skinner, 2008). The impact of transient interaction is an important area for further investigation as policymakers and service providers are starting to take seriously, comments made on social network sites about their services. For example, in 2014, a start‐up company called HealthBerry claimed to be able to draw together from across social media, comments made about specific NHS services, and received huge interest from service providers (King, 2014). Service providers are also responding to misinformation about services on social media. For example, a Facebook posting about free Calpol for children (used for minor illness) from pharmacies in the United Kingdom went viral (Buchanan, 2015), prompting NHS organizations to send clarifying information to health professionals dealing with the swell of parents' requests for free Calpol (personal communication).

## Conclusion

We set out to find out the potential for impact on health policy of the activity of lay controlled, health‐related digital social networks. We found no evidence that this social networking was resulting in mass protests about health risks or poor health care. However, there were examples, such as through Mumsnet, where sharing health care experiences through social networking led to campaigns to improve health care. These campaigns used established processes for influencing policy. Our study was limited to well established platforms where professional platform managers have considerable power to shape the direction of campaigns. It is, perhaps, more likely that social networking leading to campaigns with potential to bring about radical change, or even overwhelm or destabilize health care provision, will arise from platforms where interaction is transient.

Social networking about health raises awareness of available health care, and sensitizes individuals to their need for health care. Indirectly, this can lead to increased demand for health care which may in turn lead to changes in service provision and policy change. This has been documented for improved access to HIV treatment in South Africa, but most of the related networking was offline. Such impact will, by its nature, be relatively slow and the link between social networking and changes in health service provision difficult to detect.

## Notes

1 See example: http://en.wikipedia.org./wiki/File:StephenProfile2011.jpg.

2 For example, http://bacsytructuyen.com/, http://www.webtretho.com/, http://www.lamchame.com/
